# Co-Culture of *S*. *epidermidis* and Human Osteoblasts on Implant Surfaces: An Advanced *In Vitro* Model for Implant-Associated Infections

**DOI:** 10.1371/journal.pone.0151534

**Published:** 2016-03-16

**Authors:** Sarah Zaatreh, Katharina Wegner, Madlen Strauß, Juliane Pasold, Wolfram Mittelmeier, Andreas Podbielski, Bernd Kreikemeyer, Rainer Bader

**Affiliations:** 1 Biomechanics and Implant Technology Research Laboratory, Department of Orthopedics, University Medicine Rostock, Rostock, Mecklenburg-Western Pomerania, Germany; 2 Institute of Medical Microbiology, Virology and Hygiene, University Medicine Rostock, Rostock, Mecklenburg-Western Pomerania, Germany; University of Iowa Carver College of Medicine, UNITED STATES

## Abstract

**Objectives:**

Total joint arthroplasty is one of the most frequent and effective surgeries today. However, despite improved surgical techniques, a significant number of implant-associated infections still occur. Suitable *in vitro* models are needed to test potential approaches to prevent infection. In the present study, we aimed to establish an *in vitro* co-culture setup of human primary osteoblasts and *S*. *epidermidis* to model the onset of implant-associated infections, and to analyze antimicrobial implant surfaces and coatings.

**Materials and Methods:**

For initial surface adhesion, human primary osteoblasts (hOB) were grown for 24 hours on test sample discs made of polystyrene, titanium alloy Ti6Al4V, bone cement PALACOS R^®^, and PALACOS R^®^ loaded with antibiotics. Co-cultures were performed as a single-species infection on the osteoblasts with *S*. *epidermidis* (multiplicity of infection of 0.04), and were incubated for 2 and 7 days under aerobic conditions. Planktonic *S*. *epidermidis* was quantified by centrifugation and determination of colony-forming units (CFU). The quantification of biofilm-bound *S*. *epidermidis* on the test samples was performed by sonication and CFU counting. Quantification of adherent and vital primary osteoblasts on the test samples was performed by trypan-blue staining and counting. Scanning electron microscopy was used for evaluation of topography and composition of the species on the sample surfaces.

**Results:**

After 2 days, we observed approximately 10^4^ CFU/ml biofilm-bound *S*. *epidermidis* (10^3^ CFU/ml initial population) on the antibiotics-loaded bone cement samples in the presence of hOB, while no bacteria were detected without hOB. No biofilm-bound bacteria were detectable after 7 days in either case. Similar levels of planktonic bacteria were observed on day 2 with and without hOB. After 7 days, about 10^5^ CFU/ml planktonic bacteria were present, but only in the absence of hOB. Further, no bacteria were observed within the biofilm, while the number of hOB was decreased to 10% of its initial value compared to 150% in the mono-culture of hOB.

**Conclusion:**

We developed a co-culture setup that serves as a more comprehensive *in vitro* model for the onset of implant-associated infections and provides a test method for antimicrobial implant materials and coatings. We demonstrate that observations can be made that are unavailable from mono-culture experiments.

## Introduction

Total joint arthroplasty has been one of the most successful interventions in orthopedic surgery in recent years [1]. In Germany, approximately 350,000 primary total hip and knee endoprostheses are implanted annually [2]. However, implant-associated infections are described in 0.5% to 3% of primary and 4% to 6% of revision total hip arthroplasties [3],[4]. Despite many efforts, including modern surgery regulations, aseptic conditions, perioperative antibiotics, and antimicrobial implant surfaces, implant-associated infections cannot be completely prevented [5],[6].

The existing infection rates in orthopedic surgery are affected by the specific combination of synthetic materials, including metal alloys (e.g., Ti6Al4V) or polymers, such as bone cement, and small infection-provoking bacterial inocula [7]. After the implantation of an endoprosthesis, pathogens begin to adhere to implant surfaces and form bacterial biofilms [8]. Inside these biofilms, they are protected from the host immune defense and systemic antibiotic treatments [9]. Accordingly, bacterial adhesion to biomaterial surfaces and the formation of biofilms are important factors in the pathogenicity of microorganisms [10].

Coagulase-negative *Staphylococci*, such as *Staphylococcus epidermidis*, are the main cause of implant-associated infections, followed by other *Staphylococci*, especially *Staphylococcus aureus* [11]. The sequential order of the implant colonization by autologous cells and bacteria may cause another problem of implant-associated infections ("race for the surface") [12]. It is possible for the eukaryotic cells (e.g., human primary osteoblasts) to be restricted in their growth and colonization capability due to an initial bacterial colonization of the implant surface [13],[14]. However, even a successful initial cell growth cannot prevent bacterial infections in the long term [15].

Implant-associated infections after total joint replacement are treated by one- or two-stage revision surgery in which the infected implant is replaced [16],[17],[18]. In both cases, the healing process is supported by systemic antibiotics. Additionally, the two-stage revision involves implant removal and implantation of temporary spacers loaded with antibiotics (commonly gentamicin and vancomycin) for several weeks. This treatment enables the direct application of antibiotics to the affected tissue [19]. Considering this treatment, biocompatible and antibacterial materials for endoprostheses and spacers are highly sought after. To test new materials, improved surfaces or production and modification methods, as well as *in vitro* models, are required.

We employed an experimental test setup that allows the antimicrobial effects of biomaterials and implant surfaces to be investigated by co-cultivation of osteoblasts and bacteria. Existing test setups for this purpose often avoid establishing a co-culture due to the increased number of variables and parameters that have to be controlled in order to achieve meaningful data, and fine-tuning of experimental conditions is needed to prevent collapse of the osteoblast population, or even to enable initial adherence. Test setups that enable growth of either osteoblasts or bacteria on test materials capture only interactions between 2 components. Our setup integrates the three components osteoblasts, bacteria, and the implant surface and may provide a significant contribution to biomaterials research.

Therefore, the objective of the present study was to establish a co-culture of human primary osteoblasts and *S*. *epidermidis* to advance modelling efforts of implant-associated infections. We also aimed to investigate whether the co-culture model offers a suitable method to analyze antimicrobial implant surfaces and coatings.

## Materials and Methods

### Test Sample Discs

Titanium alloys and polymethylmethacrylate (PMMA) bone cements are common implant materials. 4 different sterile test samples were used, one of which was loaded with antibiotics: polystyrene coverslips (Thermo Fisher Scientific, Waltham, USA), titanium alloy Ti6Al4V (DOT GmbH, Rostock, Germany) and PMMA bone cement PALACOS^®^ R pure and PALACOS^®^ R loaded with gentamicin and vancomycin (Heraeus Medical GmbH, Wehrheim, Germany). The sample discs were 11 mm in diameter and 2 mm in height. We designated the pure cement as PALACOS^®^ R and the loaded form as PALACOS^®^ R+G+V. The ratio of the PALACOS^®^ R+G+V was 40 g PALACOS R^®^ with 2 g vancomycin (Lyomark Pharma GmbH, Oberhaching, Germany) and 0.5 g gentamicin. Antibiotics-loaded PMMA bone cement is often used for spacers in two-stage revisions. Polystyrene coverslips served as the control, as polystyrene is one of the most common materials for microbial and cell cultures. Plastic materials are of interest as test materials, because they are a widespread component of medical devices (for instance, implants and catheters). The roughness of all discs was approximately R_z_ = 4 μm.

### Isolation and Cultivation of Human Primary Osteoblasts

The isolation of the human primary osteoblasts (hOB) was performed according to a previously described protocol under sterile conditions [20]. The human primary osteoblasts were taken from the spongiosa of the femoral heads of patients who underwent total hip replacement. The study was approved by the Local Ethical Committee of Rostock, Germany (registration number: A2010-10), and an informed consent was signed by each patient.

The human osteoblasts were cultivated in modified Eagle’s osteogenic cell culture medium (MEM; Biochrom AG, Berlin, Germany) containing 10% fetal calf serum (FCS), 1% penicillin/streptomycin, 1% amphotericin B, and 1% HEPES buffer (all from Gibco-Invitrogen, Darmstadt, Germany) without calcium, and the osteogenic additives dexamethasone (100 mM), L-ascorbic acid (50 μg/mL), and β-glycerophosphate (10 mM) (all from Sigma-Aldrich, Munich, Germany). The osteogenic differentiation of the human primary osteoblasts was confirmed by immunohistochemical detection of the enzyme alkaline phosphatase using a fuchsin+substrate chromogen (DAKO, Hamburg, Germany). For the further tests, the isolated cells were cultured in 25-cm^2^ flasks with 8 ml of osteoblasts in the same medium but without the 1% penicillin/streptomycin and under standard cell culture conditions (5% CO_2_ and 37°C).

### Determination of Cell Viability of hOB in Mono-Culture

The osteoblasts were cultivated on the test sample discs as a monolayer for 2 and 7 days in MEM–Dulbecco’s medium without calcium or antibiotics containing 10% fetal calf serum and osteogenic additives dexamethasone, L-ascorbic acid, and β-glycerophosphate. Metabolic activity was evaluated via mitochondrial dehydrogenase activity of water-soluble tetrazolium (WST-1; Roche, Penzbeg, Germany). The cells were incubated with the WST-1 reagent for 4 hours at 5% CO_2_ and 37°C, and then the absorption was measured at 450 nm in a microplate reader (Opsys MRTM, Dynex Technologies GmbH, Denkendorf, Germany). Additionally, qualitative cell viability was measured via live/dead staining. Calcein AM fluorescence dye showed living human primary osteoblasts cells and ethidium homodimer-1 fluorescence dye tagged dead cells (Live/Dead cell viability assay, Invitrogen, Darmstadt, Germany). The fluorescence images were taken using an inverted routine microscope (Nikon Eclipse TS-100, Nikon GmbH, Düsseldorf, Germany).

### Determination of Viability of *S*. *epidermidis* in Mono-Culture

The biofilm-forming strain of *S*. *epidermidis*, RP62A (ATCC 35984; American Type Culture Collection, Manassas, USA), was used. The strain was cultured on Columbia blood agar plates (BD, Franklin Lakes, USA). For the tests, bacteria were grown to their stationary phase (37°C, microaerobic conditions) in tryptone soy broth medium (Thermo Fisher Scientific). Then, the bacterial culture was washed once in 1x PBS (4000 rpm, 10 minutes, 4°C) and adjusted to its strain-specific optical density (OD) at 600 nm to 1×10^8^ cells/ml. Afterwards, the bacterial culture was diluted to a bacterial concentration of 10^3^ CFU/ml. Bacteria were then seeded onto the different test samples and incubated over a period of 2 and 7 days at 37°C and 5% CO2 in MEM–Dulbecco’s medium without calcium or antibiotics containing 10% fetal calf serum and osteogenic additives dexamethasone, L-ascorbic acid, and β-glycerophosphate. Prior tests confirmed that the medium has no detrimental effects on bacterial growth, despite containing potentially interfering components (data not shown). Metabolic activity of biofilm-bound *S*. *epidermidis* in mono-culture was measured using WST-1. The bacteria were incubated with the WST-1 reagent for 4 hours at 5% CO_2_ and 37°C, and the absorption was measured at 450 nm using a microplate reader (SpectraMax M2, Molecular Devices, Ismaning, Germany). Viability of biofilm-bound *S*. *epidermidis* was measured qualitatively via live/dead staining (LIVE/DEAD BacLight^™^ Bacterial Viability Kit for microscopy, Thermo Fisher Scientific). The fluorescence images were taken using a fluorescence microscope (BX60 microscope, Olympus, Hamburg, Germany).

### Co-Culture of Human Osteoblasts and *S*. *epidermidis*

Human osteoblasts in the third passage (25,000 cells/ml) were transferred to a 24-well-plate format (Greiner Bio-One International AG, Kremsmünster, Austria) and cultured on the 4 different types of test samples. After 24 hours, *S*. *epidermidis* was used for mono-species infections with a multiplicity of infection (MOI) of 0.04 (25,000 cells/ml, 1000 CFU/ml). Higher bacterial concentrations would lead to a rapid decline of the osteoblast population, rendering observations of population dynamics impossible, see table B in S2 File. Subsequently, MEM–Dulbecco’s medium without calcium or antibiotics (Biochrom AG, Berlin, Germany) containing 10% fetal calf serum and osteogenic additives (see above) was used for the co-culture. Identical media were used for the *S*. *epidermidis* mono-culture and osteoblast mono-culture. For osteogenic differentiation, ascorbic acid, β-glycerophosphate, and dexamethasone were added to the medium (all from Sigma-Aldrich). The co-cultures were incubated over a period of 7 days under aerobic conditions at 37°C and 5% CO_2_. The medium was renewed 2 and 4 days after infection.

Because hOB and *S*. *epidermidis* quantification cannot be performed in one experiment, separate replicates were used. Measurements were taken after 2 and 7 days each.

### Determination of hOB Viability

The discs with adherent human primary osteoblasts were washed with 1x PBS, treated with 200 μl of 1x Trypsin/EDTA (PAA Laboratories GmbH, Cölbe, Germany) for 3 minutes under aerobic conditions at 37°C and 5% CO_2_. Finally, the cells were mechanically removed from the discs with a pipette tip (Eppendorf AG, Hamburg, Germany). The solution was transferred to a 1.5-ml Eppendorf reaction tube, centrifuged at 900 rpm for 4 minutes at 4°C, and washed with 1x PBS. Quantification of living primary osteoblasts on the test samples was performed by trypan-blue staining (Sigma-Aldrich), and subsequent counting of living cells using an Abbe-Zeiss counting cell chamber (Carl Zeiss AG, Jena, Germany) under a light optical microscope (Olympus CKX41SF, Olympus GmbH, Hamburg, Germany).

### Biofilm-Bound *S*. *epidermidis*

The sample discs were relocated to glass test tubes (Greiner Bio-One International AG, Kremsmünster, Austria) containing 1 ml of 1x PBS. *S*. *epidermidis* was removed by ultrasonic treatment for 4 minutes at 100% (device specific setting) (BactoSonic, BANDELIN electronic GmbH & Co. KG Berlin, Germany) after 2 and 7 days. The solution in the glass test tube was diluted in 1x PBS and plated onto TSB-agar plates for counting of colony-forming units after 24 hours of incubation at 37°C and 5% CO_2_.

### Planktonic *S*. *epidermidis*

The supernatant of the culture containing planktonic *S*. *epidermidis* was transferred into 15-ml centrifuge tubes (Greiner Bio-One International AG, Kremsmünster, Austria) with 1 ml of 1x PBS, and centrifuged at 4000 rpm for 10 minutes at 4°C. Subsequently, planktonic bacteria were quantified by serial dilution in 1x PBS for determination of colony-forming units on TSB-agar plates after 24 hours of incubation at 37°C and 5% CO_2_.

The pH value of the media in the three approaches was measured via pH meter (inoLab^®^ pH 720, WTW GmbH, Weilheim, Germany) after 0, 2 and 7 days (see table A in S2 File).

### Scanning Electron Microscopy (SEM)

SEM was used for evaluation of the formation of osteoblasts and bacteria on the test samples, as well as the topography and composition of the bacterial biofilm. The test samples were fixed in a 2.5% glutaraldehyde solution, then washed with 0.1 M sodium acetate buffer, and dried in an ascending ethanol series (5 min 30% ethanol; 5 min 50% ethanol; 10 min 70% ethanol; 15 min 90% ethanol; 2x 10 min 100% ethanol). Finally, the samples were exposed to critical-point drying with CO_2_ (Critical Point Dryer, Emitech, Ashford, UK), sputter-coated with gold, and investigated with a scanning electron microscope (Zeiss DSM 960A, Jena, Germany).

### Statistical Analysis

All experiments were performed with 3 biological replicates, and the results are shown as means ± standard error of the mean (SEM). Statistical significance was assessed by unpaired, 2-tailed t-test using SPSS Statistics Version 20 (IBM Corp., New York, USA). The level of significance was set at 0.05.

## Results

To elucidate the benefits of a co-culture–based test, we analyzed three experiments: (i) the mono-culture of human primary osteoblasts; (ii) the bacterial mono-culture of *S*. *epidermidis*; and (iii) the co-culture of osteoblasts and bacteria. For the mono-cultures, we used the WST-1 assay for quantification and live/dead staining for qualitative analysis. Human osteoblasts were quantified using trypan-blue staining and *S*. *epidermidis* via CFU counting both in the mono- and co-culture. Additionally, SEM imaging was employed for all 3 approaches.

### Mono-Culture of Human Primary Osteoblasts

**Fig 1:**
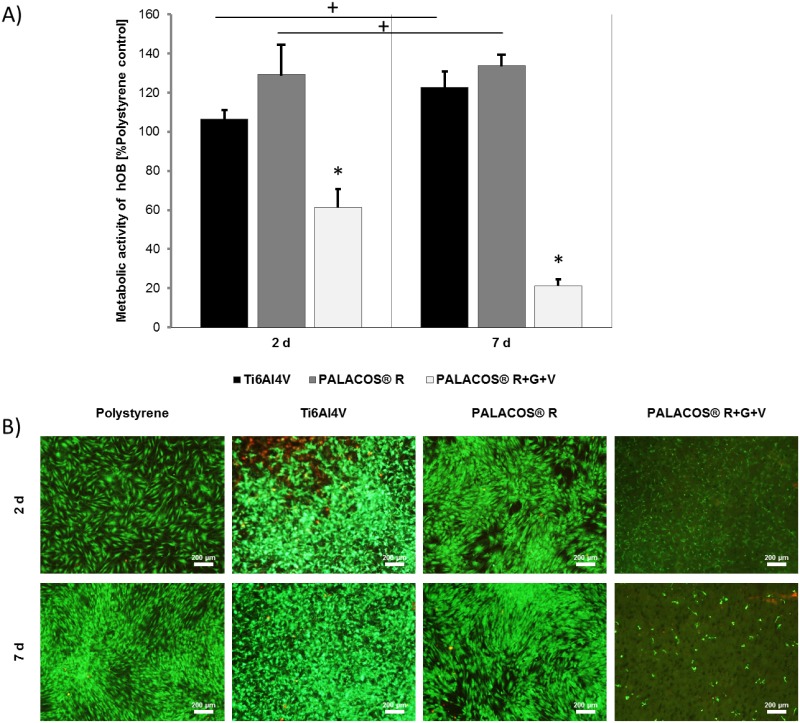
Cell viability evaluation of human primary osteoblasts in mono-culture. **A)** Metabolic activity of human primary osteoblasts (hOB) incubated for 2 and 7 days on Ti6Al4V, PALACOS^®^ R, and PALACOS^®^ R+G+V, and measured using the WST-1 assay. Values are given as percentage of the polystyrene control. PALACOS^®^ R+G+V displayed lower cell activity than the other materials on both days. (*) Denotes significance with respect to the polystyrene control at each time point, and (+) significance with respect to the same material on day 2 (n = 4, mean ± SEM). **B)** Live/dead staining of the hOB cultured in a monolayer on the test samples after 2 and 7 days of incubation. All images were taken at 40x magnification. Scale bars are 200 μm.

The metabolic activity of human primary osteoblasts was measured via the WST-1 assay (Fig 1A). There were no statistically significant differences in metabolic activity for Ti6Al4V and PALACOS^®^ R compared to the polystyrene control. In contrast, PALACOS^®^ R+G+V showed a significantly lower activity after day 2 and 7 of culture compare to the polystyrene control. Live/dead staining confirmed the results qualitatively (Fig 1B). Dead cells detached from the PALACOS^®^ R+G+V sample discs, leading to no visible staining.

### Bacterial Mono-Culture

**Fig 2:**
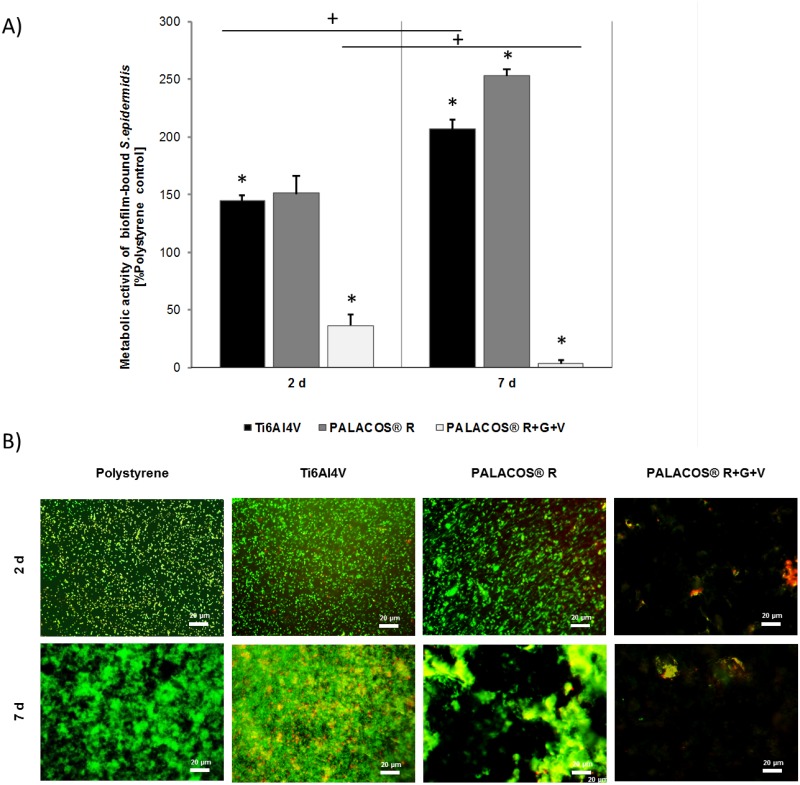
Evaluation of metabolic activity of biofilm-bound *S*. *epidermidis* in mono-culture. **A)** Metabolic activity of biofilm-bound *S*. *epidermidis* incubated for 2 and 7 days on Ti6Al4V, PALACOS^®^ R, and PALACOS^®^ R+G+V, and measured using the WST-1 assay. Values are given as a percentage of the polystyrene control. PALACOS^®^ R+G+V displayed approximately 25% and 0% activity after 2 and 7 days respectively. (*) Denotes significance with respect to the polystyrene control at each time point, and (+) significance with respect to the same material on day 2 (n = 4, mean ± SEM). **B)** Live/dead staining of the biofilm-bound *S*. *epidermidis* on the test samples after 2 and 7 days of incubation. All images were made at 400x magnification. Scale bars are 20 μm.

The metabolic activity of biofilm-bound *S*. *epidermidis* was measured via the WST-1 assay (Fig 2A). Data are shown as percentages of the polystyrene control. For Ti6Al4V and PALACOS^®^ R, the metabolic activity increased to approximately 150% on day 2 and 200–250% on day 7. On PALACOS^®^ R+G+V, the metabolic activity declined to approximately 30% on day 2 and to 0% on day 7. As in the hOB mono-culture, live/dead staining qualitatively confirmed the result (Fig 2B). For PALACOS^®^ R, the biofilm appeared in a more distinct structure, which may be a result of the difference of the material surfaces. Dead cells detached from the PALACOS^®^ R+G+V sample discs, leading to no visible staining.

### Co-Culture of Human Osteoblasts and Bacteria

**Fig 3:**
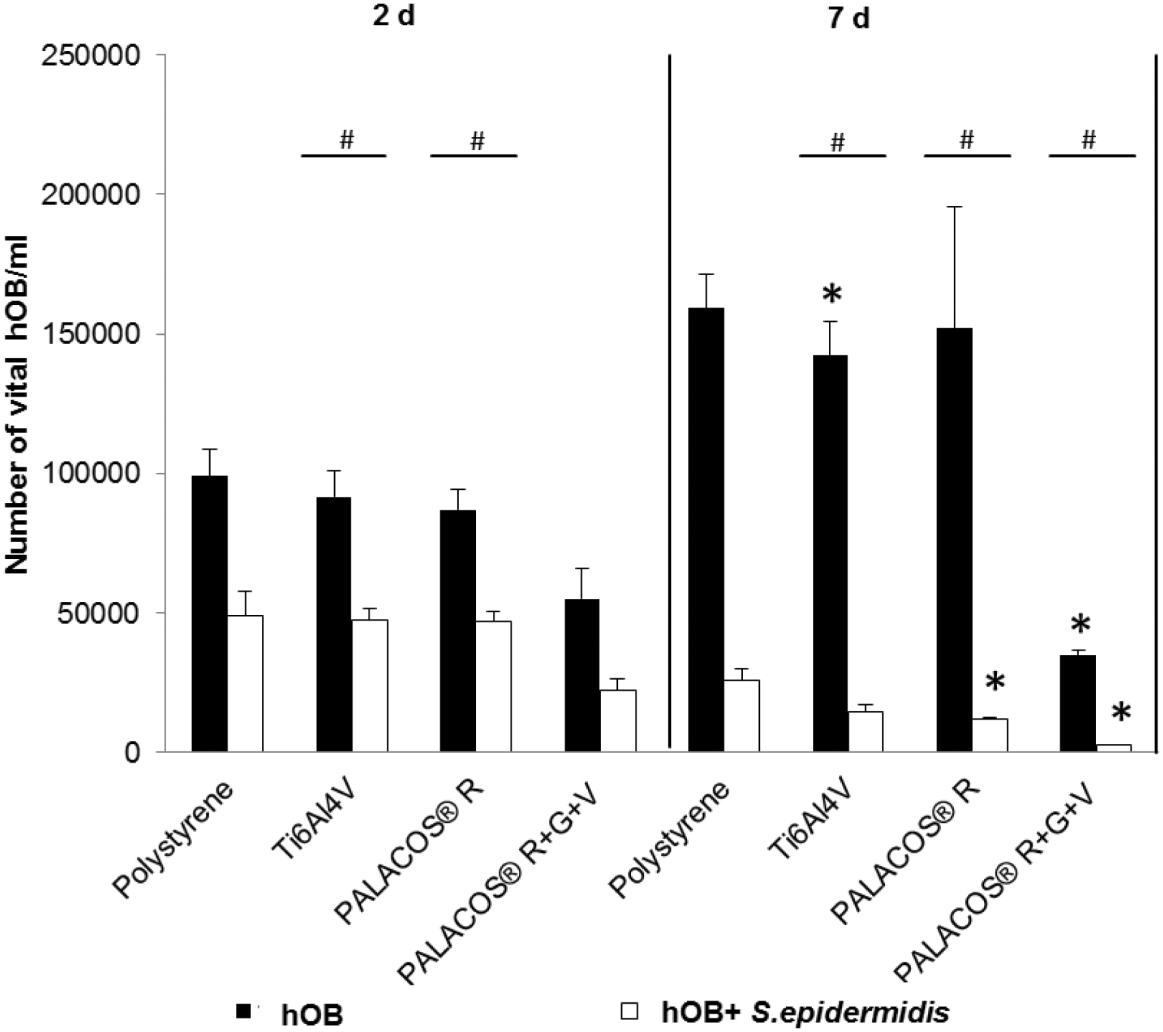
Cell viability of human primary osteoblasts in mono-culture and in co-culture. Number of viable hOB incubated on polystyrene, Ti6Al4V, PALACOS^®^ R, and PALACOS^®^ R+G+V as measured by trypan-blue staining after 2 and 7 days. All values on day 7 are significant with respect to their corresponding values on day 2. (*) Denotes significance with respect to the polystyrene control, and (#) significance with respect to the mono-culture (n = 4, mean ± SEM).

We determined the number of viable cells of hOB in the mono-culture (black bars) and the co-culture (white bars) after 2 and 7 days by trypan-blue staining (Fig 3). In the mono-culture on day 2, for Ti6Al4V, PALACOS^®^ R, and the polystyrene control, approximately 1x10^5^ viable hOB were detected. In contrast, about 5x10^4^ viable hOB were determined for PALACOS^®^ R+G+V. After 7 days of mono-culture, the amount of living hOB for Ti6Al4V, PALACOS^®^ R, and the polystyrene control had increased to 1x10^5^ (150%) compared to day 2, whereas the amount of viable cells on PALACOS^®^ R+G+V decreased to about 2,5x10^4^ (25%) viable hOB).

In the co-culture at day 2, the amount of viable cells decreased on the polystyrene control, Ti6Al4V, and PALACOS^®^ R to about 5x10^4^ viable hOB (50% compared to the hOB mono-culture). For PALACOS^®^ R+G+V, the number of viable hOB decreased to about 3,5x10^4^, 50% of the polystyrene control at day 2.

At day 7 in the co-culture, the value for the polystyrene control decreased to approximately 15% compared to the mono-culture and to 2,6x10^4^, 50% of its value from day 2. Viable hOB on Ti6Al4V and PALACOS^®^ R decreased to about 1,3x10^4^, 50% of the polystyrene control on day 7, and on PALACOS^®^ R+G+V to about 2,5x10^3^ (15%).

### Quantification of Vital Planktonic and Biofilm-Bound *S*. *epidermidis* by CFU Counting

In the mono-culture, biofilm-bound *S*. *epidermidis* grew from 1x10^6^ to 1x10^8^ CFU/ml on the non-antibiotic materials within 7 days, and showed differences in growth depending on the material. After 2 and 7 days, the number of viable biofilm-bound bacteria on PALACOS^®^ and Ti6Al4V remained unchanged. For polystyrene, the number of viable biofilm-bound bacteria had increased after 7 days of culture.

**Fig 4:**
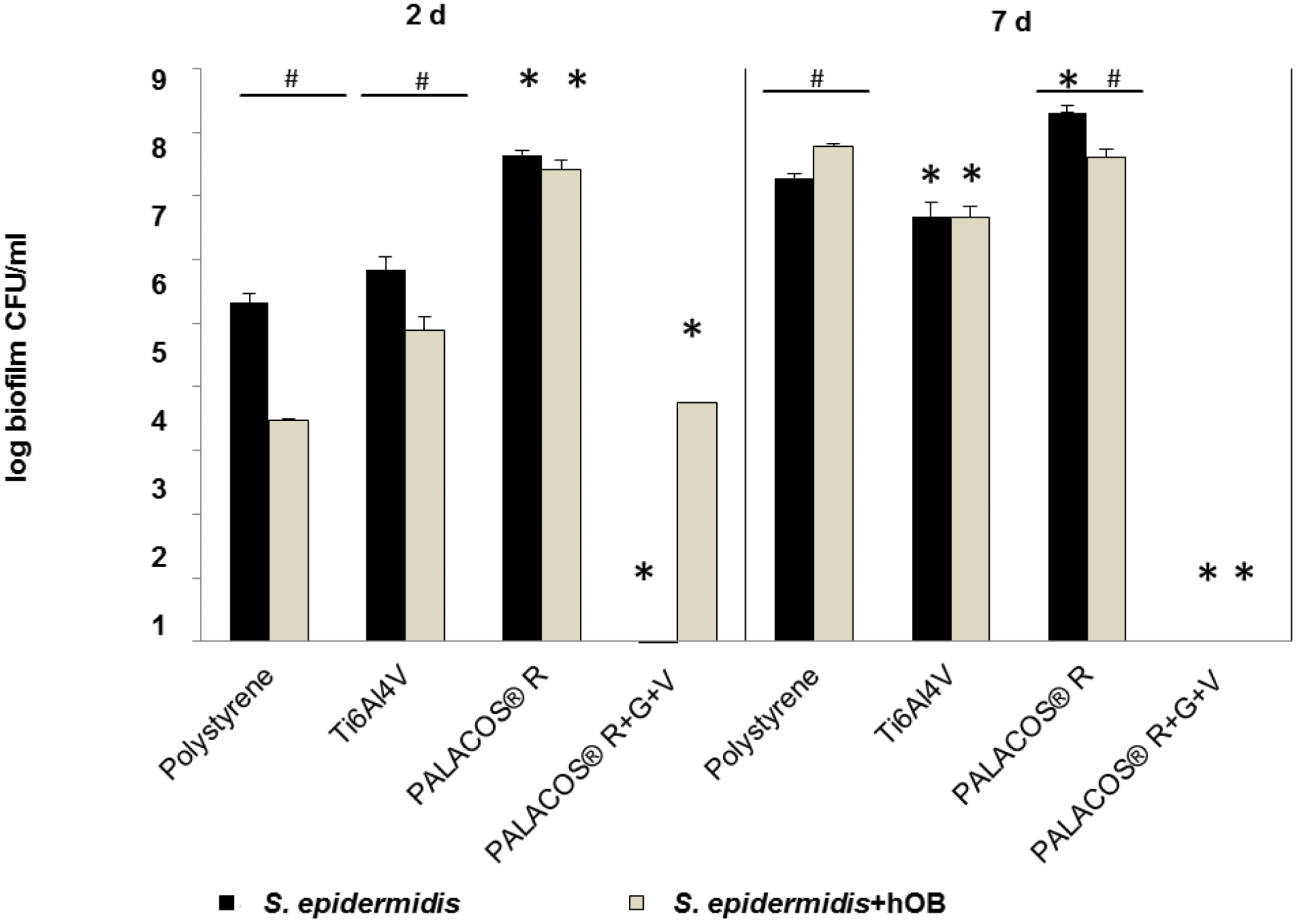
Quantification of viable biofilm-bound *S*. *epidermidis* by CFU counting. *S*. *epidermidis* mono-culture and co-culture with hOB in the biofilm on the 4 test samples (polystyrene control, TI6Al4V, PALACOS^®^ R, PALACOS^®^ R+G+V) after 2 and 7 days. PALACOS^®^ R+G+V test samples showed minimal viable bacteria except in the co-culture after 2 days. (*) Denotes significance with respect to the polystyrene control, and (#) significance with respect to the mono-culture (n = 4, mean ± SEM).

After 2 days, the number of viable biofilm-bound bacteria on polystyrene, as well as on Ti6Al4V, was decreased in the co-culture compared to the mono-culture. After 7 days in the co-culture, the number of viable biofilm-bound bacteria remained stable for the three non-antibiotic materials. In comparison to the mono-culture, the number of viable biofilm-bound bacteria ranged from 1.5x10^6^ to 1.5x10^7^ CFU/ml. For the antibiotics-loaded PALACOS^®^ R+G+V, no biofilm-bound bacteria were detected after 2 and 7 days in the mono-culture. In contrast, after 2 days of co-culture, about 1.5x10^3^ CFU/ml were observed. After 7 days of co-culture, no viable biofilm-bound bacteria were detected (Fig 4).

**Fig 5:**
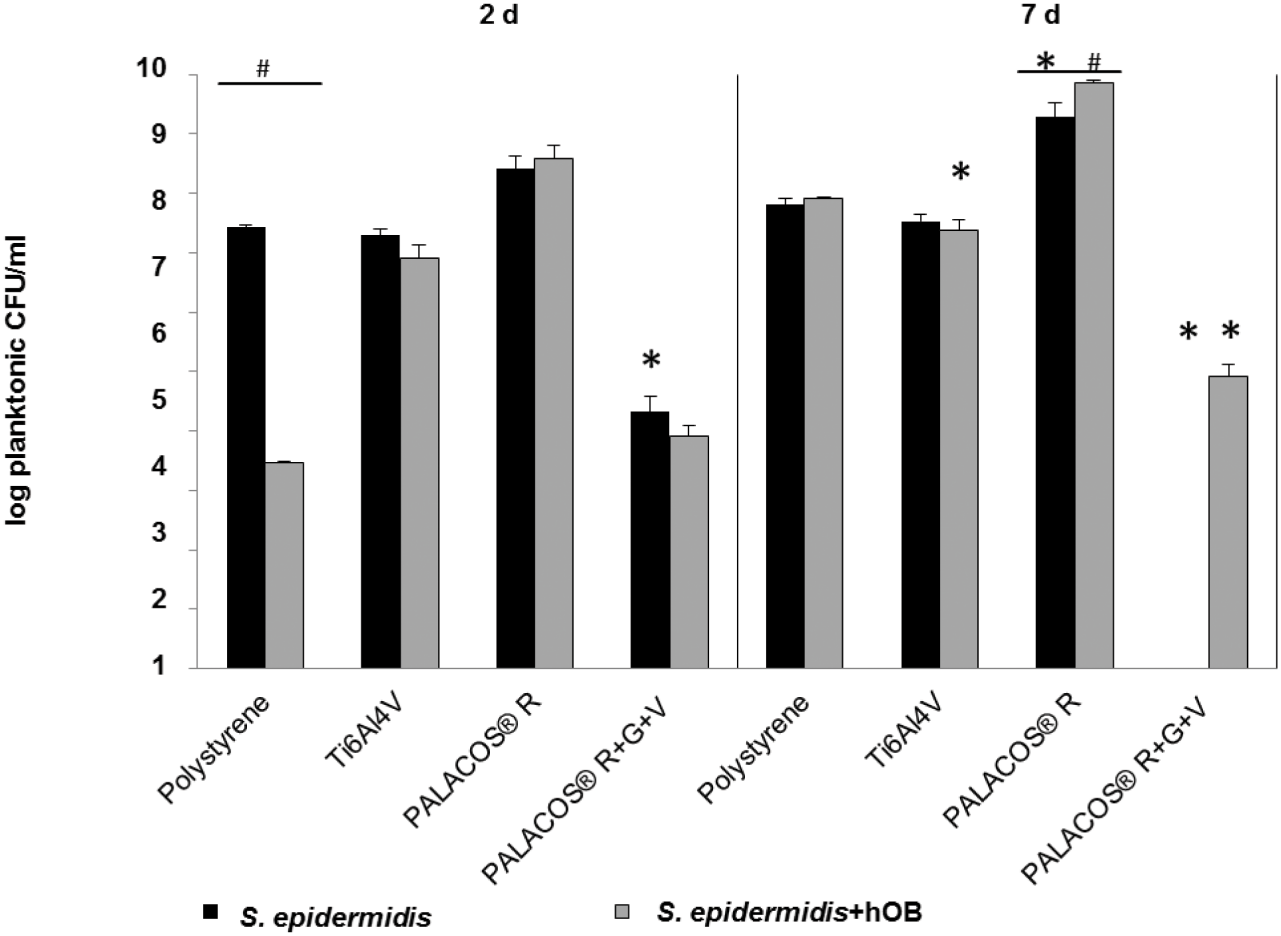
Quantification of viable planktonic *S*. *epidermidis* by CFU counting. Viable planktonic *S*. *epidermidis* were quantified in the medium after 2 and 7 days of cultivation with and without hOB on the 4 test samples (polystyrene control, TI6Al4V, PALACOS^®^ R, PALACOS^®^ R+G+V). Qualitatively, we observed the same results as for biofilm-bound *S*. *epidermidis*, except for PALACOS^®^ R+G+V where viable cells remain after 2 days in both experiments and after 7 days in the co-culture. (*) Denotes significance with respect to the polystyrene control, and (#) significance with respect to the mono-culture (n = 4, mean ± SEM).

Results for the planktonic *S*. *epidermidis* were generally similar to the CFU/ml values for the biofilm-bound with the following exceptions: Polystyrene showed increased CFU/ml values for the mono-culture and Ti6Al4V had increased values for both the mono- and the co-culture. The largest deviation between planktonic and biofilm-bound bacteria occurred for PALACOS^®^ R+G+V: There were 1x10^4^ to 1x10^5^ CFU/ml planktonic bacteria at day 2 in the mono-culture and on day 7 in the co-culture, compared to no measurable bacteria in the biofilm (Fig 5).

### Scanning Electron Microscopy

**Fig 6:**
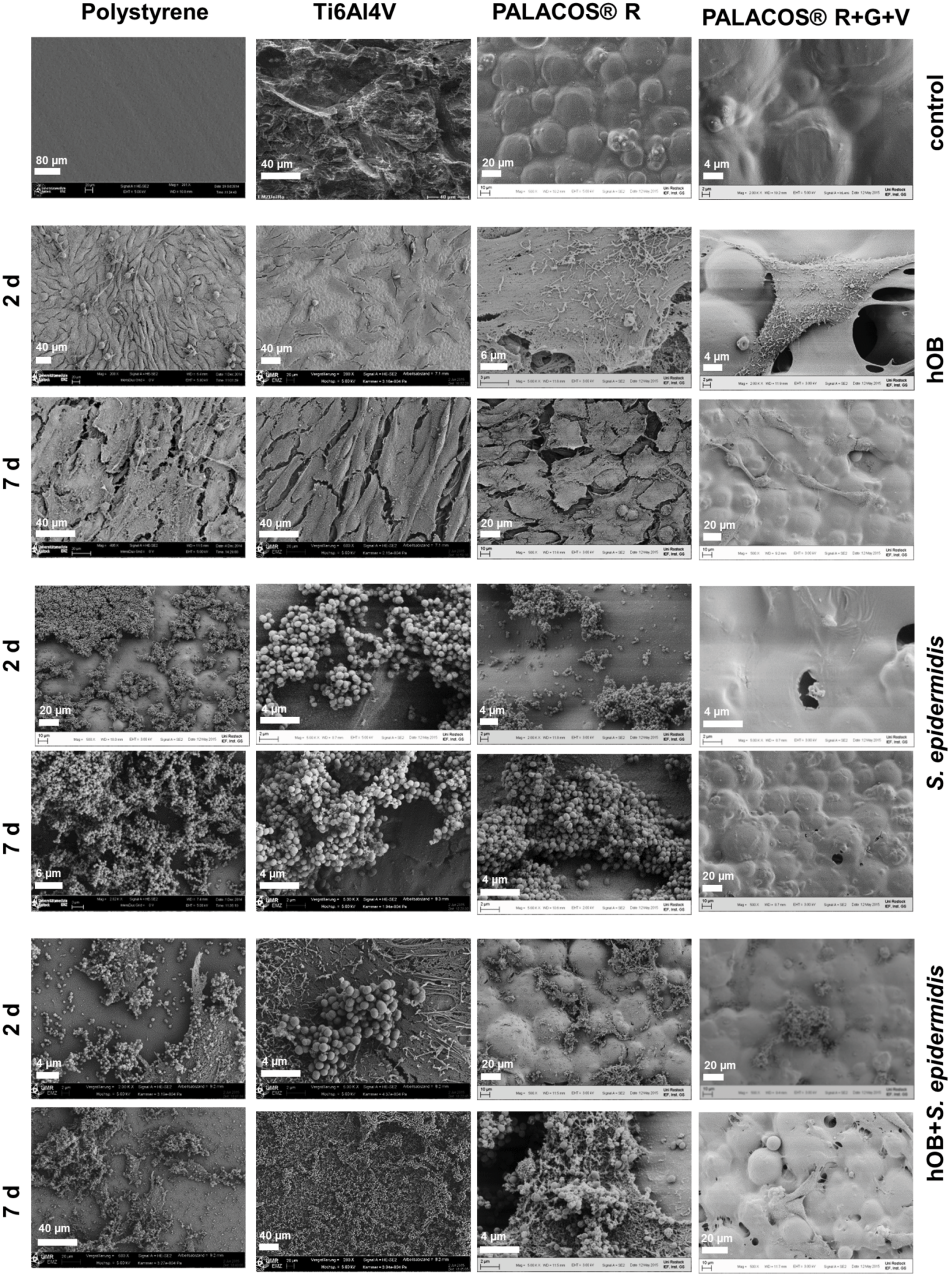
SEM images of different samples. Row 1: Test samples in medium. Rows 2 and 3: hOB mono-culture on test samples. Rows 4 and 5: *S*. *epidermidis* mono-culture on test samples. Rows 6 and 6: Co-culture of hOB and *S*. *epidermidis* on test samples. Images were taken at magnification 500x, 2000x or 2500x.

Scanning electron microscopy (SEM) images offer insight into the qualitative differences in morphology, composition, and topography of the cell complex composed of osteoblasts and bacteria on the tested surfaces. Selected images are shown in Fig 6.

The quantitative results are reflected in the SEM images: hOB free of *S*. *epidermidis* showed confluent or nearly confluent growth on all surfaces but PALACOS^®^ R+V+G (rows 2–3). The threadlike protrusions on top of the cells are microvilli, which indicate that they are viable cells[21]. Together with the typical pseudopodia formations, they can help identify hOB in the co-culture images. In the bacterial culture without hOB (rows 4–5), growth can be observed on all surfaces, except for PALACOS^®^ R+V+G, and an increased growth on day 7 compared to day 2 was observed. On PALACOS^®^ R+V+G, almost no growth was detected.

In the co-culture (rows 6–7), concurrent growth of hOB and bacteria on all surfaces was observed. The SEM images reveal that after co-culturing, *S*. *epidermidis* predominantly grew in clusters on top of the osteoblasts. After 7 days of cultivation, growth of *S*. *epidermidis* on a smaller but still significant scale on PALACOS^®^ R+V+G in the presence of hOB was noted. Furthermore, confluent growth of hOB is often interrupted and the underlying surface is visible (rows 6–7).

## Discussion

### Mono-Culture Experiments

The mono-cultures (hOB and *S*. *epidermidis* separately) served as guideline to find a working setup for the co-culture experiments, as a technical control, and for comparison of the results. We established the setup of the co-culture with the help of the mono-culture setups, most importantly number of cells for each species, type of medium, media volumes, medium renewal intervals and incubation times. Table B in S2 File shows the conditions we tested but found unsuitable, because the hOB population collapsed too quickly and therefor no meaningful measurements could be taken. The co-culture set-up where hOB and *S*. *epidermidis* were seeded simultaneously did not lead to a successful culture due to hOB not being able to adhere to the sample disc surface in time [12],[13],[22]. It can be presumed that seeding the *S*. *epidermidis* before the hOB would lead to the same result, due to the bacteria claiming the available surface.

In the mono-culture with primary human osteoblasts, the cells grow as well on the materials without antibiotics (Ti6Al4V, PALACOS^®^ R) as on the control. The antibiotics-loaded surface (PALACOS^®^ R+G+V) showed inhibitory effects on the osteoblasts after 2 days, which was even more pronounced after 7 days. According to Isefuku *et al*., Gentamicin at high concentrations inhibits cell proliferation of hOB *in vitro* and is speculated to be interfering with repair processes *in vivo* [23].

The mono-culture of *S*. *epidermidis* displayed uninhibited growth on polystyrene, Ti6Al4V, and PALACOS^®^ R, as expected. PALACOS^®^ R+G+V prevented growth of the bacteria on its surface and, therefore, decreased the cell number of planktonic bacteria to nearly zero.

The decreased viability of the human primary osteoblasts in the co-culture experiment can be explained by a combination of the following factors:

Bacteria form cell contacts and directly interfere with hOB proliferation, possibly via the production of bacterial toxins. Both species are in competition for nutrients from the growth medium, available surface space, and in driving the pH value towards their favored regimen [24]. More acidic pH values are favorable for *S*. *epidermidis*, while human cells prefer neutral or slightly basic pH values. Pathogenic *S*. *epidermidis* are more capable of driving the pH value in their favor, as osteoblasts rely on the bloodstream and other support to maintain a favorable pH environment [25]. The pH-values for the mono and co-culture experiments can be found in table A in S2 File.

The results of the mono-culture are expected and we view them as confirmation of the soundness of our setup. We consider this an important step to increase confidence in the fact that observations in the co-culture are not artifacts, due to a flawed set-up.

### Co-Culture Experiments

From the results of the co-culture experiments we want to highlight three distinct observations:

1. PALACOS^®^ R+G+V is significantly less effective in the presence of osteoblasts, S. epidermidis survives in the biofilm on day 2
2. In the presence of osteoblasts, a substantial planktonic population survives in the test with PALACOS^®^ R+G+V, even to day 7
3. The antibiotic effect of PALACOS^®^ R+G+V on the hOB is stronger than the positive effect of reducing the S. epidermidis population

On day 2, we observed significant survival of *S*. *epidermidis* on the antibiotics-loaded PALACOS^®^ R+G+V in the presence of hOB. Not only are *S*. *epidermidis* evolved to flourish in the environment of human cells, it can be speculated that hOB offer a secondary surface, thus shielding and diminishing the effects of the antibiotics diffusing from the bone cement’s surface, and this shielding is effective enough to increase bacterial viability.

On day 7, the planktonic bacterial population in the co-culture had increased by 2 orders of magnitude from the initial value, in contrast to the mono-culture in which no planktonic bacteria were measurable. A speculative explanation could be that with the surface being hostile the bacterial population transfers into the solution, aided by the slowed down effect of the antibiotics in the presence of the hOB.

As shown above, PALACOS^®^ R+G+V had detrimental effects on hOB in mono-culture and removed the *S*. *epidermidis* in mono-culture entirely. We were able to answer the question, whether the net effect of the antibiotics on the hOB is positive or negative. Viability of hOB was roughly 10% of the initial population on day 7 on PALACOS^®^ R+G+V, lower than in the hOB mono-culture. The detrimental effects of the antibiotics outweigh the reduction of the bacterial population in our experiments.

These three observations cannot be derived from mono-culture experiments and reveal interactions only present in the co-culture. The increased modelling power is instrumental in revealing otherwise unavailable results. It can be assumed, that overestimation of antibiotics’ effectiveness and underestimation of a material's cytotoxicity may occur in mono-culture tests more so than in co-culture based tests.

Mono-cultures can only capture the interaction of human cells or bacteria with surfaces, not the interaction of human cells and bacteria, which can be expected to be highly non-trivial. These effects can arise from interactions involving all three components (human cells, bacteria and implant surfaces and can only be captured by a co-culture model. Our study highlights that a co-culture model can show un-observable effects, which can have important implications for subsequent research. The potential “shielding” effect of hOB with the bone cement’s antibiotics and the transition to a planktonic bacteria population in the face of the implant surface [10] could be investigated. The majority of current *in vitro* studies use comparable approaches (e.g. static in-vitro mono-cultures) [26]. These approaches can be extended to co-cultures in order to detect potentially compounding effects.

However, our present study is limited in some respects. As an *in vitro* model, it is inherently limited in terms of the provided environment, e.g., no bone formation processed, and no immune system, surrounding bloodstream, or supportive tissue. In a next step to advance this field of research, we would ideally aim for a stable balance of the hOB and the bacterial population. This could be approached by more frequent media renewal (selectively removing a portion of the bacterial population), the use of flow-cell reactors (compare, for instance, Lee *et al*.) or by adding supportive components of the immune system or antibiotics to counterbalance the bacterial growth and pathogenicity [14], [27], [28].

We could only explore a limited number of experimental parameters, most importantly incubation time, initial number of hOB and bacteria, time until infection, different growth media, and hOB cell lines, as well as bacterial species and strains. A further improvement could lie in retrieving gene expression levels [26] as an additional data source to gain insight into the immune response and a more detailed picture of the on-going processes.

## Conclusion

The objective of this study was to develop a co-culture model of the onset of implant-associated infection. Such models are essential to find biocompatible materials and surfaces with antimicrobial properties, as well as for testing antibiotics.

We were able to identify a working setup that led to successful co-culture of hOB and *S*. *epidermidis* on different test materials. We performed mono-culture experiments as a basis for comparison and technical guideline to develop the co-culture setup. Our experiments have revealed results unavailable to mono-culture–based approaches, offering a more comprehensive model. The setup can be used to form new hypotheses and guide experiments to investigate implant-associated infections and to test implant materials in a more realistic setting. The shielding effect of the hOB with the bone cement’s antibiotics or the transition of the bacteria population to a planktonic one in the face of an antibiotics loaded implant surface can be examined.

The next steps to improve our co-culture model would be to acquire data for longer incubation times and variation of experimental parameters and the use of continuous flow culture techniques.

## Supporting Information

S1 File Experimental results. This excel file contains the data for all the experimental results: *S*. *epidermidis* mono-culture, the primary human osteoblast mono-culture and the co-culture, each for days 2 and 7.
(XLSX)

S2 File Supplementary tables. Table A: pH values in the three approaches for after 0, 2 and 7 days, Table B: tested co –culture conditions.
(DOCX)
