# Supplementary material for: Co-Culture of S. epidermidis and Human Osteoblasts on Implant Surfaces: An Advanced In Vitro Model for Implant-Associated Infections
Source: PLoS One. 2016 Mar 16;11(3):e0151534. doi: 10.1371/journal.pone.0151534 (PMC4794246; doi:10.1371/journal.pone.0151534)
Supplement: S2 File — Table A: pH values in the three approaches for after 0, 2 and 7 days, Table B: tested co –culture conditions. (DOCX) [file pone.0151534.s002.docx]

**Supplementary table A. pH values in the three approaches for** **polystyrene, Ti6Al4V, PALACOS® R and PALACOS® R+G+V samples after 0, 2 and 7 days**

| **Mono-culture of human primary osteoblasts** | **0 d** | **2 d** | **7 d** |
| --- | --- | --- | --- |
| **Media MEM +10% FCS** | 7,95 | 7,89 | 7,92 |
| **Ti6Al4V** | 7,92 | 7,94 | 7,67 |
| **Polystyrene coverslips** | 7,96 | 8,06 | 7,74 |
| **PALACOS® R** | 8,04 | 8,31 | 7,76 |
| **PALACOS® R+G+V** | 8,01 | 8,41 | 7,58 |
|  |  |  |  |
| **Mono-culture of *S. epidermidis*** | **0 d** | **2 d** | **7 d** |
| **Media MEM +10% FCS** | 8,1 | 7,83 | 7,96 |
| **Ti6Al4V** | 7,98 | 7,95 | 5,69 |
| **Polystyrene coverslips** | 7,91 | 7,94 | 5,66 |
| **PALACOS® R** | 7,99 | 8,2 | 8,24 |
| **PALACOS® R+G+V** | 8,04 | 8,24 | 8,12 |
|  |  |  |  |
| **Co-culture of human osteoblasts and bacteria** | **0 d** | **2 d** | **7 d** |
| **Media MEM +10% FCS** | 7,94 | 7,89 | 7,92 |
| **Ti6Al4V** | 7,88 | 7,85 | 6,66 |
| **Polystyrene coverslips** | 7,98 | 7,91 | 6,47 |
| **PALACOS® R** | 8,03 | 8,31 | 7,7 |
| **PALACOS® R+G+V** | 8,07 | 8,35 | 7,79 |

**Supplementary table B. Tested co –culture conditions**

|  | **hOB count** | ***S. epidermidis count*** | **time of inoculation** | **media  volumes** | **media  renewal  intervals** | **incubation  time** |
| --- | --- | --- | --- | --- | --- | --- |
| **discarded setups** | 2500  cells/ml | 100 CFU/ml | same time | 500µl | none | 10 days |
|  | 10000 cells/ml | 1000 CFU/ml | same time | 500µl | day 4 | 10 days |
|  | 25000 cells/ml | 1000 CFU/ml | 24 h after hOB | 1000µl | day 4 | 7 days |
| **final setup** | 25000 cells/ml | 1000 CFU/ml | 24 h after hOB | 1000 µl | day 2, 4 | 7 days |
